# Arthroscopic excision of intra-articular sub-periosteal osteoid osteoma of elbow: a case report

**DOI:** 10.1051/sicotj/2018021

**Published:** 2018-06-29

**Authors:** Tarun Goyal, Naveen Pandita, Souvik Paul

**Affiliations:** Department of Orthopaedics, AIIMS, Virbhadra Marg, Rishikesh, 248201 India

**Keywords:** Elbow, Elbow stiffness, Osteoid osteoma, Arthroscopy

## Abstract

We are presenting a unique case of a sub-periosteal osteoid osteoma involving coronoid fossa in a 25-year-old male. He was symptomatic for 2 years and his presentation mimicked mono-articular inflammatory arthritis. His plain radiographs were normal and the computed tomogram confirmed features of a sub-periosteal osteoid osteoma. He was treated with arthroscopic excision of the lesion. Pain relief was noticed immediately after the surgery and maintained at latest follow up of 1 year.

## Introduction

**Figure 1:**
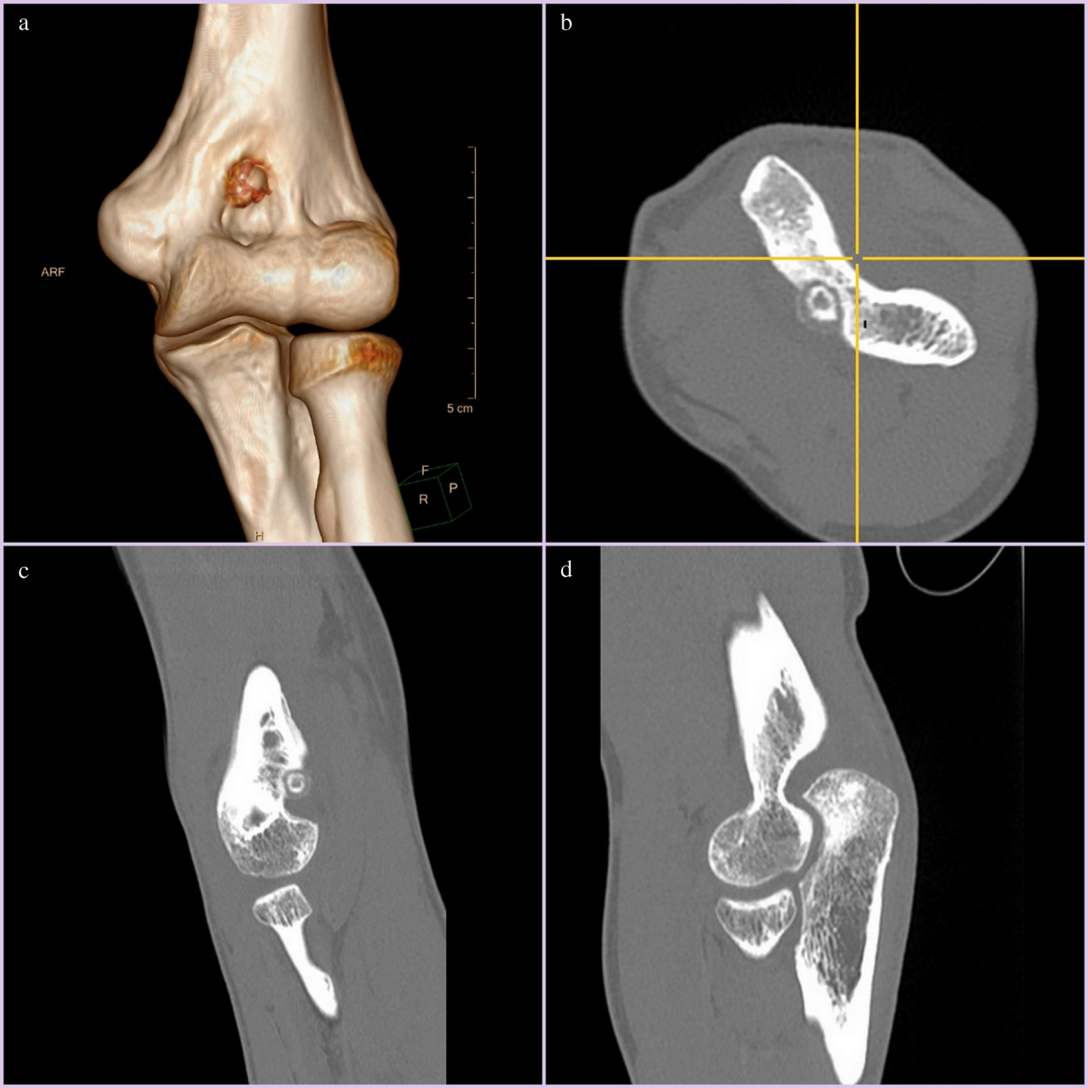
Computed tomography images showing the lesion in coronal 3D reconstruction (a), axial (b), and sagittal (c) views. (d) Shows an otherwise normal elbow joint.

Osteoid osteoma is a common benign lesion of bone. It is a bone-forming tumour and is most commonly located in the diaphysis of long bones. About half of them occur in bones of lower limb. Osteoid osteoma involving humerus is relatively rare [1]. Typically, it presents with nocturnal pain that responds to nonsteroidal anti-inflammatory drugs, especially salicylates. This typical presentation may be absent in osteoid osteomas occurring in intra-articular location [2–4]. Osteoid osteoma can be subperiosteal, cortical and cancellous. The classical radiographic appearance of cortical osteoid osteoma is a radiolucent nidus with surrounding sclerosis or cortical thickening. In contrast, intra-articular osteoid osteoma has little or no reactive sclerosis. Intra-articular osteoid osteomas are very rare and form only up to 5–12% of all osteoid osteomas [2,5–7]. Then they reach the joint surface they may present with signs and symptoms of mono-articular arthritis, including swelling, pain and stiffness. Symptoms are often non-specific and may often be confused with other causes of mono-articular involvement such as inflammatory or infectious arthritis [2,4,8,9]. Thus diagnosis is often delayed. Very few cases of intra-articular osteoid osteoma of elbow joint have been described in literature [2–4,7–22]. Sub-periosteal location of an elbow osteoid osteoma has not been described before. They have been mostly treated with open excision of the lesion. There are only limited reports on arthroscopic removal of osteoid osteoma of elbow [10–14] and shoulder [23,24]. This case presents a unique case of arthroscopic removal of a sub-periosteal osteoid osteoma involving coronoid fossa and presentation mimicking mono-articular inflammatory arthritis Figure 1.

## Case

A 25-year-old male, right hand dominant and manual labourer by occupation came to our hospital with history of pain and stiffness of right elbow for last 2 years. Pain was mainly worse after periods of rest and after waking up in the morning, with no history of nocturnal exacerbations. Pain relived only partially with salicylates and other non-steroidal anti-inflammatory medications. He had no other musculoskeletal complaints or any other significant past medical history. He had previously sought consultation in other places and various diagnoses such as lateral epicondylitis, mono-articular rheumatoid involvement.

**Figure 2:**
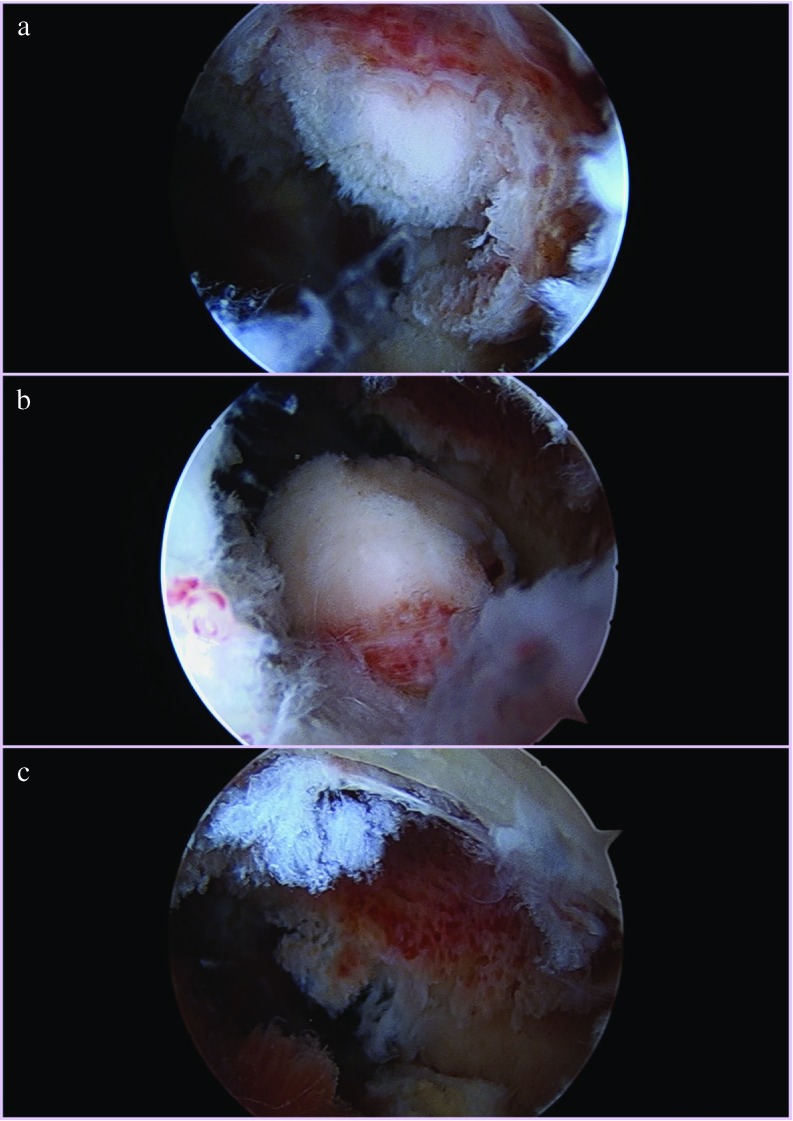
Arthroscopic images showing identification of the lesion after removal of synovium and thin cortical shell (a) removal of the nidus and surrounding sclerosis, (b) and clear base after removal of the lesion.

Physical examination revealed no swelling or fullness around the elbow. There was mild tenderness along the anterior aspect of elbow. There was restriction of both flexion and extension with elbow range of motion from 30 to 90^°^. Supination and pronation were normal. Plain radiographs were normal. Computed tomography (CT) with 3D reconstruction revealed a radio-dense ring measuring about 4 mm in the coronoid fossa of humerus. It had a radiolucent nidus measuring about 2 mm in diameter. There was a thin bony shell in the anterior aspect of the lesion. These findings were consistent with a diagnosis of a sub-periosteal osteoid osteoma. A decision of arthroscopic excision was made Figure 2.

Elbow arthroscopy was carried out under general anesthesia in lateral decubitus position. Standard proximal anteromedial viewing (anterior and 2 cm proximal to medial epicondyle) and proximal anterolateral instrumentation portals (anterior and 2 cm proximal to lateral epicondyle) were used for elbow arthroscopy. A 2.7-mm, 30° arthroscope was used. The lesion was localised in the coronoid fossa. It appeared as a hyperaemic bony protuberance covered with hypertrophied synovium. Synovium was shaved with an arthroscopic shaver. Thin cortical shell over the lesion was removed with an arthroscopic shaver. The nidus was exposed and it shelled out easily upon manipulation with a curette. It was delivered through the anteromedial arthroscopic portal and sent for histopathology. The base of the lesion was cleaned with curette and burr. Normal bone deeper to the base of the lesion was not removed and posterior cortex was not perforated. No other arthroscopic releases were done. Intraoperative imaging was net required as the lesion was large and easily visible upon insertion of arthroscope.

Histopathology findings of the nidus were consistent with an osteoid osteoma. There was an interlacing network of osteoid and bony trabeculae with some mineralization. Histopathology of the synovium only showed features of non-specific chronic inflammation. Gram stain and culture results were negative. His complete blood counts, erythrocyte sedimentation rate and C-reactive protein were within normal limits.

**Figure 3:**
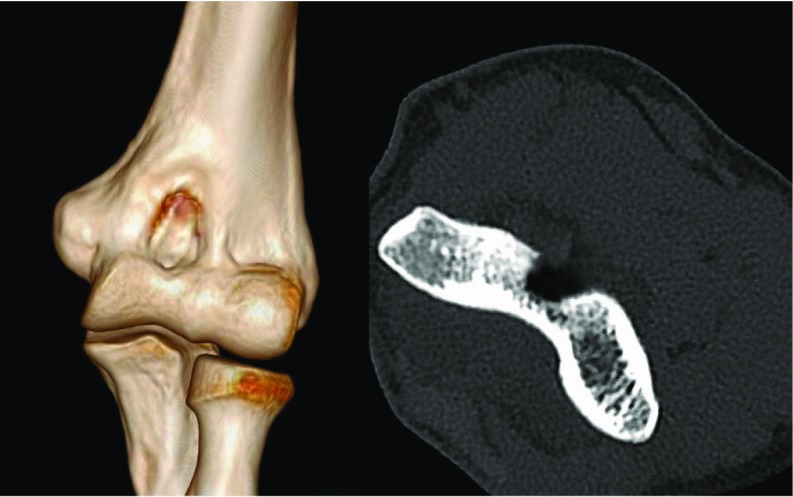
Post-operative computed tomography images showing base after removal of the lesion.

Pain relief after the procedure was remarkable. He had no pain after 3 days and could resume his work. At latest follow up of 1 year patient is completely pain-free. He has full range of motion of the elbow (0–150°, Figure 3). 

## Discussion

Osteoid osteomas may be frequently missed on a plain radiograph as approximately 25% of them may not be seen [11]. This may be further difficult in intra-articular osteoid-osteomas because of lack of osteo-sclerosis, peculiar anatomy and overlap of bones. CT scan with fine cuts is the best investigation as it can define exact location, size of nidus and can show zone of surrounding sclerosis. Intra-articular osteoid osteoma generally do not evoke as profound osteo-sclerosis as cortical osteoid osteomas [4]. This may be related to their location in the area rich in cancellous bone. Sclerosis was only mild in this case, extending to about 2 mm circumferentially around the lesion and the nidus was very clearly visible on the CT scan. Adjacent bone appeared normal and the lesion was very well demarcated from the normal surrounding bone. No periosteal reaction was seen. Since the diagnosis was certain by clear visualisation of a nidus on CT scan, patient was planned for elbow arthroscopy further studies such as magnetic resonance imaging or bone scintigraphy, which would be indicated only in case of a doubtful diagnosis [15].

Clinically differential diagnosis could be mono-articular rheumatoid arthritis, tubercular arthritis or osteochondritis dissecans. CT scan was suggestive of an osteoid osteoma, but a possibility of chronic osteomyelitis should also be considered [4]. Intraoperative tissue cultures were negative, ruling out chronic osteomyelitis. CT scan may show an irregular inner surface of nidus in chronic osteomyelitis compared to a smooth inner surface in an osteoid osteoma [4,16]. Due to symptoms mimicking mono-arthritis the patient had visited in departments of general medicine and rheumatology prior to coming to us. A synovial sample from a patient with intra-articular osteoid osteoma may have some features similar to the synovium in rheumatoid arthritis. Sanchis-Alfonso et al emphasised that specific features of synovium in rheumatoid arthritis, such as, papillary synovial pattern, hyperplastic epithelium, characteristic vascular changes and deeper infiltration lymphoid cells should be sought [7].

Some aspects of arthroscopic technique may merit discussion. There were florid intra-articular adhesions in this case as described by Bhatia [14]. We used standard proximal anteromedial and proximal antero-lateral portals. There was no need for a 70° arthroscope or accessory portals for visualisation [14]. Capsular releases are not required for gaining motion. Other treatment options could have been an open excision or a CT scan guided radiofrequency ablation. In intra-articular tumours, radiofrequency should be used with caution because of a risk of causing osteonecrosis of bone.

In elbow joint osteoma has been described in olecranon fossa [17,4], trochlea [18] or capitulum [18,10,15], proximal radial [15,19], proximal ulna [4,9,20], coronoid fossa [7,8,14,21,22], medial epicondyle [15]. Thus, coronoid fossa is a relatively common site to be involved in elbow joint. All of them have been described to be cancellous osteoid osteomas [23], A sub-periosteal location of osteoid osteoma in elbow joint has not been described before.

Thus this case highlights the difficulty in diagnosis of a case of mono-articular elbow pain and stiffness. Due to absence of specific features of osteoid osteoma, diagnosis may be delayed in juxta-articular lesions. A neoplastic aetiology should always be considered in differential diagnosis of a mono-articular joint pain, stiffness and synovitis. Evaluation with a CT scan or a MRI should be carried out. Relief of symptoms is generally very dramatic after resection of osteoid osteoma.

## Conflict of interest

The authors declare that they have no conflicts of interest in relation to this article.
